# A hypervirulent *Acinetobacter baumannii* strain has robust anti-phagocytosis ability

**DOI:** 10.1186/s12866-024-03264-x

**Published:** 2024-04-01

**Authors:** Yan Li, Mohan Jv, Yuan Zhuang, Xu Zhao, Xiaoxiong Hu

**Affiliations:** 1 Division of Infectious Diseases, Yichun People’s Hospital, Yichun, Jiangxi Province China; 2 Institute of Antibiotics, Huashan Hospital, Fudan University, Shanghai, China; 3 Department of Infectious Diseases, People’s Hospital of Rizhao, Ri Zhao, Shandong Province China; 4 Department of Infectious Diseases, Huashan Hospital Fujian Campus, Fudan University, Fuzhou, Fujian Province China

**Keywords:** *Acinetobacter baumannii*, Community-acquired pneumonia, Hypervirulent, Anti-phagocytosis, Type VI secretion system

## Abstract

**Background:**

*Acinetobacter baumannii* (*A. baumannii*) is associated with both hospital-acquired infections (HAP) and community-acquired pneumonia (CAP). In this study, we present a novel CAP-associated *A. baumannii* (CAP-AB) strain causing severe pneumonia in an afore healthy male patient without underlying conditions. Subsequently, we investigated the pathogenicity and immunogenicity of this CAP-AB strain using a mice pneumonia model.

**Results:**

A 58-year-old male patient with no underlying conditions experienced worsening symptoms of a productive cough, sputum, and fever that developed acutely, in just 24 h. The diagnosis was severe community-acquired pneumonia (CAP) and type-1 respiratory failure. An *A. baumannii* strain was isolated from his sputum and blood cultures. To gain a deeper understanding of the rapid progression of its pathology, we utilized the CAP-associated *A. baumannii* strain YC128, a previously obtained hospital-acquired pneumonia *A. baumannii* (HAP-AB) strain YC156, and a highly virulent *A. baumannii* control strain LAC-4 to construct a mouse pneumonia model, and subsequently compared the mortality rate of the three groups. Following inoculation with 10^7^ CFU of *A. baumannii*, the mortality rate for the YC128, LAC-4, and YC156 groups was 60% (6/10), 30% (3/10), and 0%, respectively. The bacterial burden within the pulmonary, liver, and spleen tissues of mice in the YC128 group was significantly higher than that of the YC156 group, and slightly higher than that of the LAC-4 group. Pathological analysis of lung tissue using HE-staining revealed that the inflammatory pathological changes in mice from the YC128 group were significantly more severe than those in the YC156 group. Additionally, CT scan images displayed more pronounced inflammation in the lungs of mice from the YC128 group compared to the YC156 group. Local levels of cytokines/chemokines such as IL-1β, IL-6, TNF-α, and CXCL1 were assessed via RT-qPCR in lung tissues. In comparison with the YC156 strain, the highly virulent YC128 strain induced the expression of proinflammatory cytokines more rapidly and severely. Furthermore, we examined the in vitro anti-phagocytosis ability of YC128 and YC156 strains against mice peritoneal macrophages, revealing that the highly virulent YC128 isolate displayed greater resistance to macrophage uptake in contrast to YC156. Results from Whole Genome Sequencing (WGS) indicated that YC128 harbored a complete type VI secretion system (T6SS) gene cluster, while YC156 lacked the majority of genes within the T6SS gene cluster. The other virulence-related genes exhibited minimal differences between YC128 and YC156. Drawing from previous studies, we postulated that the T6SS is linked to the hypervirulence and robust anti-phagocytic ability of YC128.

**Conclusions:**

This article reports on the isolation of a novel hypervirulent CAP-AB strain, YC128, from a severe CAP patient. The results demonstrate that this CAP-AB strain, YC128, is capable of inducing fatal pneumonia and extrapulmonary dissemination in a mouse pneumonia model. Moreover, this highly virulent CAP-AB strain exhibits significantly stronger anti-phagocytic abilities compared to the HAP-AB YC156 strain. Genome sequencing comparisons reveal that the heightened hypervirulence and enhanced anti-phagocytosis abilities observed in YC128 may be attributed to the presence of the T6SS.

## Background

*Acinetobacter baumannii* (*A. baumannii*) has been increasingly recognized as a conventional pathogen that accounts for an array of hospital-associated infections, including pneumonia (HAP), bloodstream infections, urinary tract infections, and meningitis [1, 2]. In the past decade, the emergence of multidrug-resistant (MDR) strains of *A. baumannii*, which cause hospital-acquired infections, has become a critical challenge for physicians [3, 4]. Furthermore, *A. baumannii* has been reported to be responsible for both community-acquired pneumonia (CAP) and bacteremia [5, 6]. Compared to other hospital-acquired pneumonia (HAP) counterparts, community-acquired *A. baumannii* pneumonia (CAAP) progresses more rapidly and is associated with a higher mortality rate [7, 8]. Despite the distinct nature of strains between CAP-*A. baumannii* (CAP-AB) and HAP-*A. baumannii* (HAP-AB), there is little known about the pathogenesis and immunogenesis between these two isotypes [9, 10]. As such, to develop more effective treatment strategies for these patients, additional studies are required to gain a deeper understanding of the pathogenicity and immunogenicity of CAP-AB*.*

Here, we present a novel *A. baumannii* strain, namely YC128 (Yichun Hospital 128), which was isolated from a clinical case of CAP in Jiangxi Province, China. We investigated the pathogenicity and immunogenicity of this CAP-AB strain in parallel with a previously obtained HAP-AB strain, YC156 (Yichun Hospital 156), and a highly virulent *A. baumannii* control strain LAC-4 [11], using a mice pneumonia model. We collected data on pulmonary bacterial burden, pathological changes, CT scan images, and cytokine levels in the lungs of these mice models. The findings demonstrate that the newly isolated CAP-AB strain, YC128, exhibits hypervirulence in contrast to the HAP-AB strain, YC156. YC128 induced fatal pneumonia and bacteremia, leading to significantly increased bacterial loads in various tissues, heightened lung pathogenesis, and altered cytokine levels compared to YC156. Furthermore, YC128 significantly impeded the phagocytic abilities of peritoneal macrophages, suggesting it to be at least one of the mechanisms contributing to the robust virulence of the YC128 strain. Genome sequencing (GSW) results reveal that the observed hypervirulence and enhanced anti-phagocytosis ability of YC128 may be attributed to the presence of the type VI secretion system.

## Results

### Case history

A 58-year-old male patient visited the emergency department of Yichun Hospital, Jiangxi Province, China, in 2021. He had experienced worsening symptoms of productive cough, sputum, fever, chills, and dyspnea that developed within 24 h. The physical examination revealed a respiratory rate of 40 breaths per minute and a body temperature of 39.6℃. He had been in good health prior to this episode and had no history of chronic diseases, drug use, smoking, or alcohol consumption. The laboratory results showed a white blood cell count of 4.8 × 10^9^/l with 87.6% neutrophils and C-reactive protein > 200 mg/l. Arterial blood gas analysis at room air revealed the following values: pH 7.485, pCO2 25.4 mmHg, pO2 58.9 mmHg. The diagnosis was severe community-acquired pneumonia (CAP) and type-1 respiratory failure. A CT scan revealed pronounced inflammation and complete consolidation in the right upper lobe. On the third day after admission, sputum and blood culture results indicated the presence of *A. baumannii* bacteria, the bacterial burden of blood and sputum were both 4 + , no definite quantitative results. Treatment for this patient consisted of Imipenem/Cilastatin 1 g ivgtt q8h plus amikacin 0.6 g ivgtt qd. Following ten days of treatment, the patient's symptoms of productive cough and sputum had alleviated, and the body temperature returned to normal. The treatment regimen lasted for 15 days, during which the patient's symptoms steadily improved, and subsequent sputum and blood culture tests returned negative results. A follow-up CT scan showed a significant reduction in inflammation and consolidation in the right upper lobe. Consequently, the case exhibited marked improvement. The strain isolated from the blood culture of this patient was designated as CAP-AB strain YC128.

### Antimicrobial susceptibility of the two *A. Baumannii* strains

The minimal inhibitory concentrations (MICs) of YC128 and YC156 were determined using the agar dilution method, and the antimicrobial susceptibility results of the two strains are listed in Table 1. The data revealed that the CAP-AB strain YC128 was sensitive to all testing antimicrobial agents including families of aminoglycosides, fluoroquinolones, cephalosporins and carbapenems. On the other hand, the HAP-AB strain YC156 was a carbapenem-resistant *A. baumannii* (CRAB), exhibiting sensitivity only to Tigecycline (1 μg/ml) and Colistin (0.5 μg/ml) (Table 1).

**Table 1:** Antimicrobial susceptibility of the two *A. baumannii*

Antimicrobial agent	MIC (μg/ml)	
	CAP-AB YC128	HAP-AB YC156
Ceftazidime	2	128
Cefepime	2	128
Ciprofloxacin	0.125	> 32
Amikacin	0.5	> 256
Meropenem	0.5	> 32
Imipenem	0.5	> 32
Piperacillin-tazobactam	8	> 256
Cefoperazone-sulbactam	2	128
Tigecycline	0.125	1
Colistin	0.25	0.5

### YC128 induced significantly increased mortality rate in mice

**Fig. 1:**
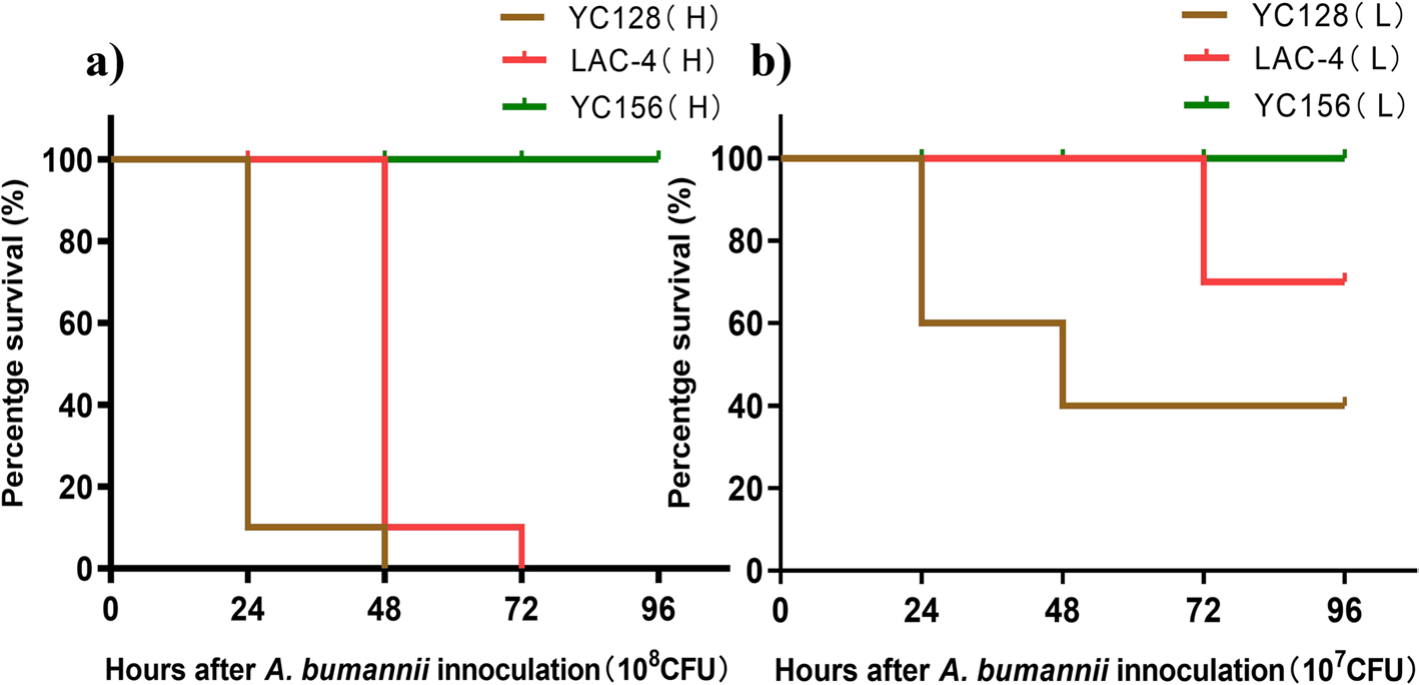
YC128 induced significantly increased mortality rate in mice. Sixty mice were intranasally inoculated with *A. baumannii* in 50 μl of saline, with each group 10 mice, in addition, five mice was intranasally inoculated with 50 μl saline as negative control. **a** Every mouse in group YC128(H), LAC-4(H) and YC156(H) was inoculated with 10^8^ CFU *A. baumannii* respectively. **b** Every mouse in group YC128(L), LAC-4(L) and YC156(L) was inoculated with 10^7^ CFU *A. baumannii* respectively. (H: high *A. baumannii* suspension, 10^8^ CFU; L: low *A. baumannii* suspension, 10^7^ CFU)

In the YC156 group, no mice died from intranasal inoculation with either 10^7^ CFU or 10^8^ CFU of bacteria. In contrast, within the YC128 and LAC-4 groups, all mice (10/10) that were intranasally inoculated with a bacterial suspension of 10^8^ CFU died within 48 h. When a bacterial suspension of 10^7^ CFU was administered, no participants (0/10) died in the YC156 group, 60% (6/10) died in the YC128 group, and 30% (3/10) died in the LAC-4 group. No mice died in the saline control group. Figure 1 illustrated the survival rate of mice inoculated with different *A. baumannii* strains. The mice mortality in the YC128 group was significantly higher than in the YC156 group (*P* < 0.01). Furthermore, when inoculated with 10^7^ CFU of bacteria, the mice mortality rate in the YC128 group exceeded that in the LAC-4 group (60% vs. 30%). The survival curve analysis of the YC128 group and the LAC-4 group showed borderline significance (*P* = 0.074).

### YC128 induced significantly higher bacterial burdens in tissues

**Fig. 2:**
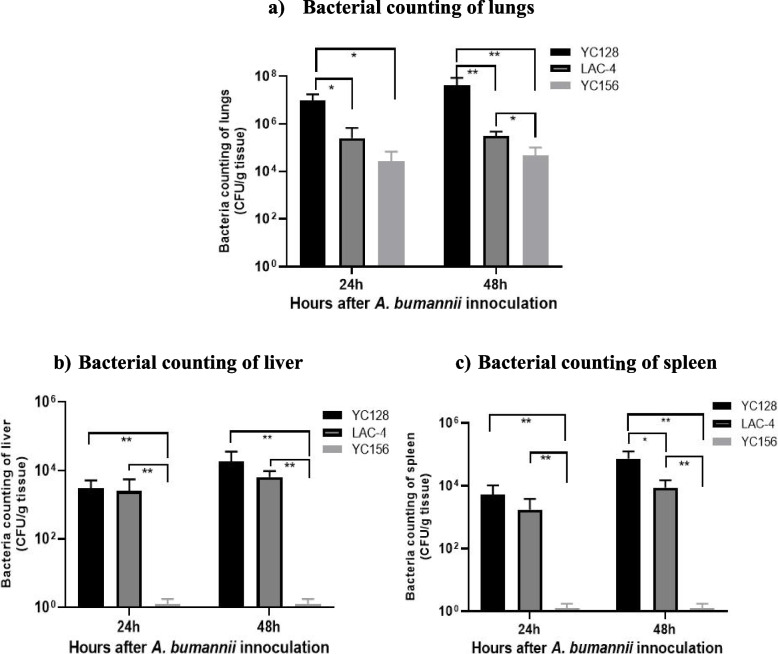
YC128 induced significantly higher bacterial burdens in tissues. The bacterial burden of YC128, LAC-4 and YC156 in mice pneumoniae model. Twenty-four mice were intranasally inoculated with 10^7^ CFU *A. baumannii* in 50 μl of saline, with each group 8 mice. Four mice were sacrificed at 24 h and 48 h after inoculation in each group. The lungs, liver and spleen from three mice were conducted quantitative culture. **a** Bacterial counting of lungs. **b** Bacterial counting of liver. **c** Bacterial counting of spleen

Twenty-four mice were intranasally inoculated with *A. baumannii*, with each group comprising 8 mice. In addition, 4 mice were intranasally inoculated with 50 μl of saline as a control. The bacterial loads of the inoculants were 5.0 × 10^7^ CFU for YC128, 4.5 × 10^7^ CFU for LAC-4, and 3.2 × 10^7^ CFU for YC156. To compare the tissue bacterial burdens of the three groups, four mice were sacrificed at 24 h and 48 h after inoculation in each group. Lungs, livers, and spleens were harvested, homogenized, and cultured on LB agar dishes, and bacterial colony-forming data were analyzed. At 24 h post-inoculation, the pulmonary bacterial burden in the YC128 group was 9.8 ± 7.9 × 10^6^ CFU/g tissue, for LAC-4 it was 2.5 ± 4.3 × 10^5^ CFU/g tissue, and for YC156 it was 2.7 ± 4.2 × 10^4^ CFU/g tissue. At 48 h post-inoculation, the pulmonary bacterial burden for YC128 was 4.3 ± 4.4 × 10^7^ CFU/g tissue, for LAC-4 it was 3.2 ± 1.7 × 10^5^ CFU/g tissue, and for YC156 it was 4.8 ± 5.5 × 10^4^ CFU/g tissue (Fig. 2a). The liver bacterial burden for YC128 and LAC-4 was 3.0 ± 0.7 × 10^3^ CFU/g tissue and 2.5 ± 2.6 × 10^3^ CFU/g tissue at 24 h, and 1.8 ± 1.1 × 10^4^ CFU/g tissue and 6.5 ± 3.1 × 10^3^ CFU/g tissue at 48 h (Fig. 2b). Meanwhile, the spleen bacterial burden for YC128 and LAC-4 was 5.3 ± 5.0 × 10^3^ CFU/g tissue and 1.8 ± 1.8 × 10^3^ CFU/g tissue at 24 h, 7.3 ± 5.1 × 10^4^ CFU/g tissue and 8.8 ± 6.3 × 10^3^ CFU/g tissue at 48 h (Fig. 2c). In contrast, the liver and spleen bacterial burdens for the YC156 group remained at 0 CFU/g tissue throughout the experiment. The lung, liver, and spleen bacterial burdens remained at 0 in the saline control group. The pulmonary, liver, and spleen bacterial burdens in the YC128 and LAC-4 groups were significantly higher than in the YC156 group. The tissue bacterial burdens of YC128 were consistently slightly higher than those of the LAC-4 group. At 48 h, the pulmonary bacterial burden of YC128 was significantly higher than in the LAC-4 group (*P* = 0.003), along with the spleen burden (*P* = 0.05). This suggests that the CAP-AB strain YC128, in comparison with the HAP-AB strain YC156, is a highly virulent strain, and the virulence of YC128 is slightly stronger than that of the highly virulent *A. baumannii* control strain LAC-4.

The inflammatory pathological changes of the YC128 infected mice were significantly more severe than the YC156 group.

**Fig. 3:**
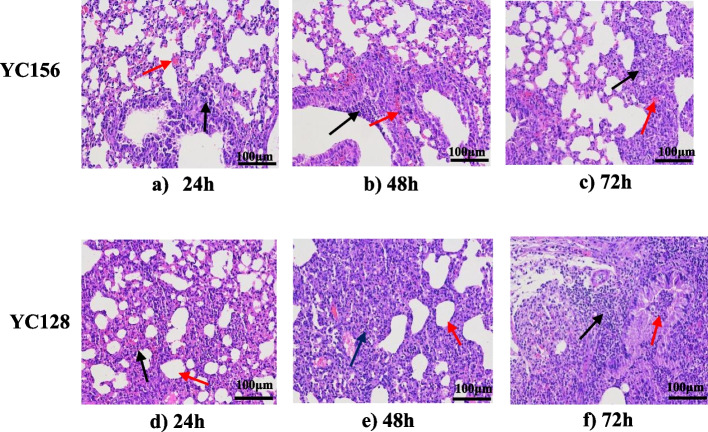
The inflammatory pathological changes of the CAP-AB infected mice were significantly more severe than the HAP-AB group. Pulmonary pathology of CAP-AB YC128 and HAP-AB YC156 group mice. **a** 24 h after YC156 inoculation. Moderate edema in pulmonary interval (red arrow), some lymphocytes and neutrophils invasion (black arrow). **b** 48 h after YC156 inoculation. Focal infiltration of lymphocytes and neutrophils in bronchial epithelium (black arrow), protein effusion with inflammatory cell infiltration in some alveolus (red arrow). **c** 72 h after YC156 inoculation, some of the alveolus atrophy and lung consolidation (black arrow), focal intra alveolar hemorrhage (red arrow). **d** 24 h after YC128 inoculation. Most alveoli atrophy, lung consolidation (black arrow), the alveoli fused into large alveoli (red arrow). **e** 48 h after YC128 inoculation. Lung consolidation, destruction of alveolus, mononuclear and neutrophils invasion (black arrow), some alveolis fused into large alveoli (red arrow). **f** 72 h after YC128 inoculation. Lung consolidation, destruction of alveolus (black arrow) with a large number of mononuclear, lymphocytes and neutrophils invasion (red arrow)

Pathological analysis of the lungs by HE-staining revealed that the inflammatory pathological changes in mice of the YC128 group were significantly more severe than that in the YC156 group. In the YC128 group, the histopathology results demonstrated that the pulmonary interstitium of the mice exhibited moderate edema, accompanied by moderate invasions of lymphocytes and neutrophils into the pulmonary cavity. There was also moderate alveolus destruction and lung consolidation (Fig. 3a, b and c). Conversely, within the YC156 group, histopathologic changes displayed severe congestion and edema at 24 h after inoculation (Fig. 3d). At 48 h and 72 h, the experimental findings indicated the occurrence of lung consolidation, necrosis, and alveolus destruction. Concurrently, a significant number of mononuclear, lymphocytes, and neutrophils invaded the pulmonary and alveolar cavities (Fig. 3e and f).

### YC128 induced more severe pneumonia that is proved by chest CT scan

**Fig. 4:**
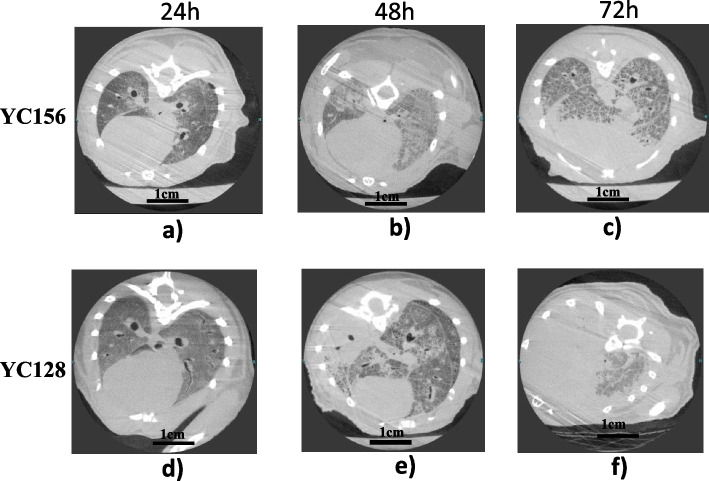
YC128 induced more severe imaging changes shown in chest CT scan. **a** 24 h after YC156 inoculation. Slight patchy opacities in both lungs. **b** 48 h after YC156 inoculation. Moderate patchy opacities in both lungs. **c** 72 h after YC156 inoculation. Patchy opacities alleviation in both lungs. **d** 24 h after YC128 inoculation. Slight patchy opacities in both lungs. **e** 48 h after YC128 inoculation. Complete consolidation was present in the whole left lung and local patchy opacities in the right lung. **f** 72 h after YC128 inoculation. consolidation appeared in bilateral lungs like “white lung”

The chest CT scan images of theYC128 and YC156 mice are shown in Fig. 4. In the YC128 group, The CT scans displayed slight patchy opacities in both lungs at 24 h (Fig. 4a), followed by increased patchy opacities in both lungs at 48 h (Fig. 4b). Subsequently, the images showed patchy opacities almost returned to normal at 72 h (Fig. 4c). Conversely, in the YC156 group, the image exhibited slight patchy opacities in both lungs at 24 h (Fig. 4d). By 48 h, severe inflammation and complete consolidation were observed in the entire left lung, alongside localized patchy opacities in the right lung (Fig. 4e). The inflammation worsened progressively, leading to consolidation in both lungs resembling a "white lung" appearance by 72 h (Fig. 4f).

### YC128 induced more rapid and severe pro-cytokines expressions in the lung

**Fig. 5:**
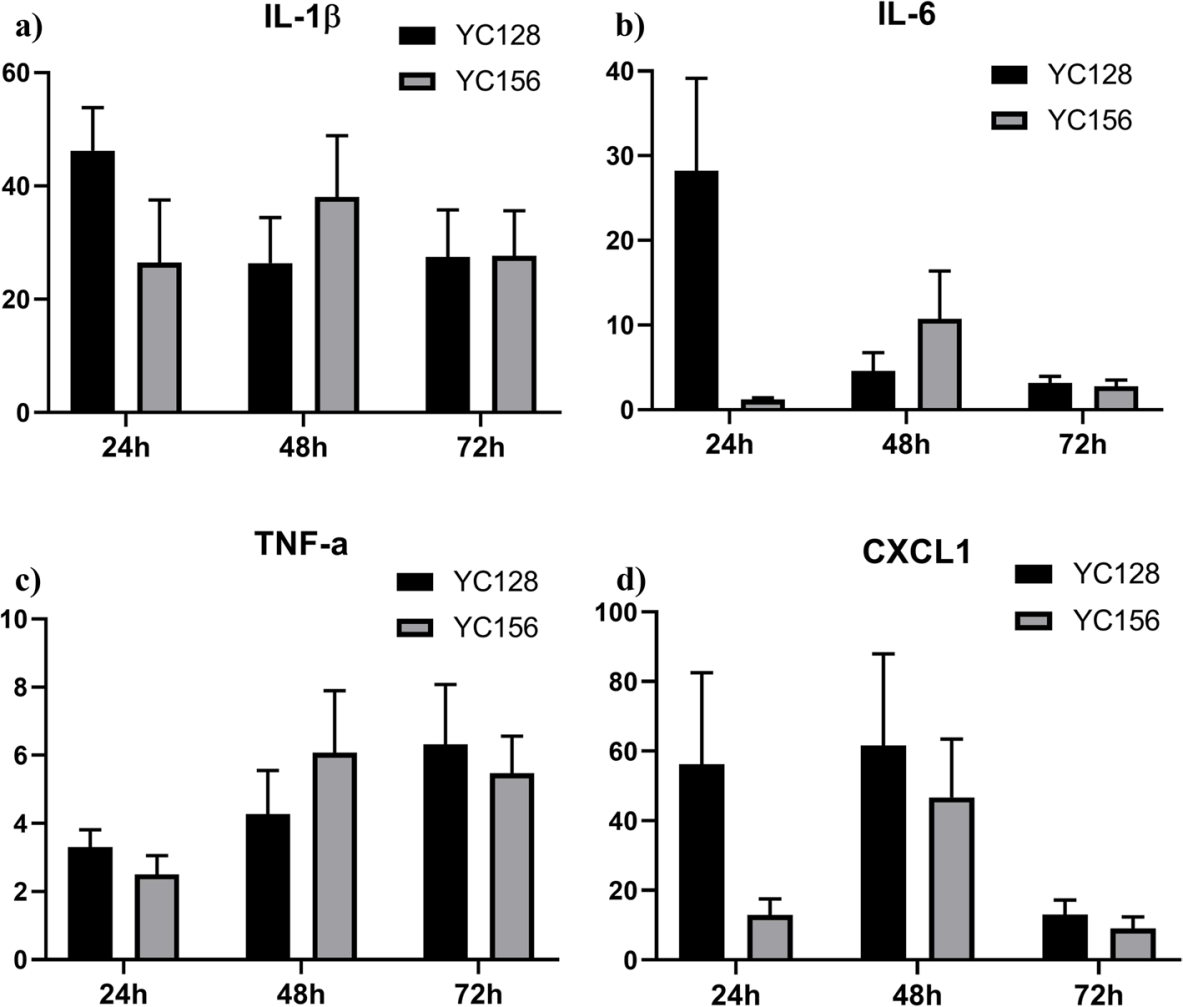
YC128 induced more rapid cytokines expressions in the lung. The local level in lung of cytokines/chemokine were detected by RT-qPCR. **a** IL-1β. **b** IL-6. **c** TNF-α. **d** CXCL1

The local (lung) levels of cytokines/chemokines (IL-1β, IL-6, TNF-α, and CXCL1) were detected by RT-qPCR, and the cytokines/chemokines expressions in the lung are shown in Fig. 5. The designated time points were 0 h, 24 h, 48 h, and 72 h post-inoculation. Regarding the IL-1β expression level in the lung of the two groups, group YC128 reached its peak at 24 h, exhibiting a 48-fold increase compared to the baseline at 0 h. In contrast, group YC156 only increased by 19-fold, a significantly lower increase than the YC128 group (*P* = 0.020). At 48 h post-inoculation, group YC156 continued to rise, reaching a 40-fold increase. On the other hand, the IL-1β expression level in group YC128 decreased to a 27-fold increase. Subsequently, the expression levels decreased to 28-fold for both groups at 72 h. However, the maximum expression level in group YC128 was 8-fold higher than in YC156 (Fig. 5a). Similar expression patterns of IL-6 and CXCL1 were observed in the two groups. The expression levels of YC128 reached their peak at 24 h, significantly higher than those of the YC156 group. For IL-6, group YC128 reached its peak at 24 h, exhibiting a 23-fold increase, whereas group YC156 increased by only 1.2-fold (*P* = 0.020). In group YC156, the peak level was reached at 48 h (fivefold), which was 18 times lower than the peak in group YC128 (Fig. 5b). As for CXCL1, group YC128 reached its peak at 24 h (98-fold), while group YC156 increased by 12-fold (*P* = 0.036). The peak level for group YC156 appeared at 48 h (30-fold), resulting in a 68-fold difference between the peaks of the two groups (Fig. 5d). The difference in TNF-α expression level peaks between the two groups was not significant. In the YC156 group, it increased by 866-fold at 48 h, and in the YC128 group, it increased by 922-fold at 24 h compared to the baseline level at 0 h (Fig. 5c).

### YC128 showed significant stronger in vitro anti-phagocytosis ability than that of YC156

**Fig. 6:**
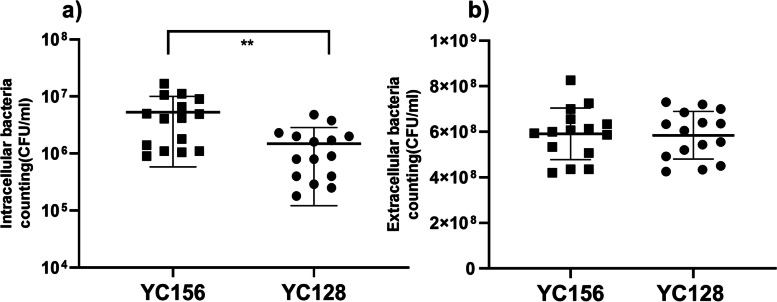
YC128 showed significant stronger in vitro anti-phagocytosis ability than that of HAP-AB strain. Peritoneal macrophages isolated from 5 mice and each mouse macrophages were distributed into 6 wells. Then, 20 μl per well of YC156 and YC128 bacterial suspension were added into the cell culture. After two hours later, the cell culture medium was collected to quantify the extracellular bacterial number. Then the wells were washed using ice-cold PBS and sterile water added into wells to lysis macrophage. Two hours later, the suspension was cultured to quantify the intracellular bacterial number. **a** Intracellular bacterial number. **b** Extracellular bacterial number

Each well was supplemented with 20 μl of YC128 and YC156 bacterial suspension, each at a specific concentration, into peritoneal macrophages isolated from 5 mice. After two hours, the macrophage culture medium was collected, and then the wells were treated with sterile water to lyse the macrophages. The resulting suspension was subsequently cultured to quantify the intracellular bacterial count. The findings demonstrated that the intracellular bacterial count within macrophages of YC128 was significantly lower than that of YC156 (1.5 ± 1.4 × 10^6^ CFU/ml vs. 5.3 ± 4.7 × 10^6^ CFU/ml, *P* = 0.0054) (Fig. 6a). The previously collected cell culture medium was assayed to determine the extracellular bacterial count. The results indicated that the extracellular bacterial count was similar between the two groups (Fig. 6b).

### Characterization of antimicrobial resistance and virulence genes of YC128 and YC156

**Fig. 7:**
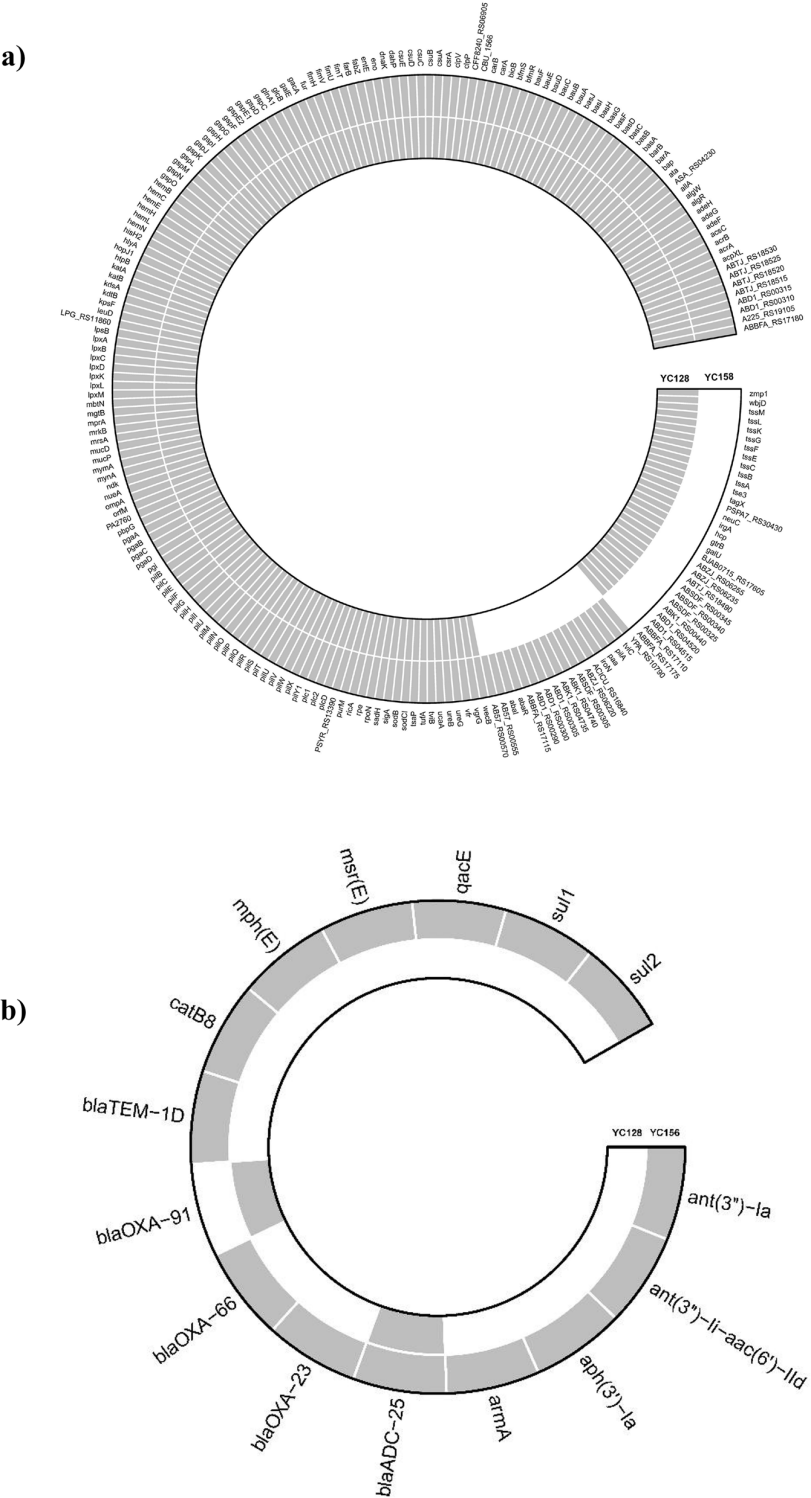
The Comparisons of Whole Genome Sequencing between YC128 and YC156. **a** Virulence related genes. **b** Antimicrobial resistance genes

Comparing the results of Whole Genome Sequencing (WGS) for YC128 and YC156, it was observed that 169 virulence-related genes, including Acinetobacter trimeric autotransporter (Ata) and Type IV pili (TFP) ect., were nearly identical between the two strains [12]. Additionally, 30 virulence-related genes were exclusively identified in YC128 but not in YC156. Among these, 11 genes were related to capsules [13], and there was also a cluster of genes associated with the Type VI secretion system (T6SS) [14] (Fig. 7a). Remarkably, YC128 contained a complete set of T6SS genes (13 in total) as well as genes encoding T6SS secreted effectors [15]. On the other hand, YC156 exhibited only one gene (T6SS tip protein VgrG) from the T6SS gene cluster [16]. Most of the T6SS genes and T6SS secreted effector genes were absent in YC156 due to this deficiency. This indicates that YC128 possesses the ability to express T6SS, whereas YC156 lacks this capability. Thus, it can be deduced that T6SS plays a pivotal role in the hypervirulence of YC128. The WGS results also revealed that YC128 had no acquired antimicrobial resistance (AMR) genes except for the chromosome carrying β-lactamase genes (blaADC-25 and blaOXA-91). In contrast, YC156 harbored 16 AMR genes, including β-lactamase genes such as blaADC-25, blaOXA-23, blaOXA-66, blaOXA-91, and blaTEM-1D, as well as genes related to sulfamide resistance (Sul1) and aminoglycoside resistance (ant(3'')-Ia and armA) [17] (Fig. 7b).

## Discussion

In 1993, Bick et al. reported an unusual case of pneumonia caused by *A. baumannii* acquired in the community. The patient was a 74-year-old Korean woman with no documented underlying medical risk factors. This case represented an early case report of Community-Acquired *A. baumannii* Pneumonia (CAAP) [18]. From then on, several community-acquired *A. baumannii* cases have been reported [19–21]. This disease progresses rapidly, providing a short window for physicians to administer appropriate treatment. However, there have been few case reports of CAAP in mainland China [22–24]. We suppose that the reason is the vast majority of doctors in China are not familiar with the specific bacteria and relevant diseases.

Most of the reported cases of CAAP were associated with underlying medical conditions, including alcoholism, smoking, chronic obstructive pulmonary disease (COPD), and diabetes mellitus. Another characteristic clinical presentation of CAAP was a fulminant course, with acute onset of dyspnea, cough, and fever that rapidly progresses to respiratory failure and shock [7, 21]. The clinical presentation of our patient was similar to those described in previous reports. However, our patient had no medical history of chronic disease and lacked recognized risk factors such as alcoholism, smoking, and COPD, etc. It was an unusual case of Community-Acquired *A. baumannii* Pneumonia (CAAP), but the underlying condition of this patient was similar of the case reported in 1993 [18] and in 2020, Zhejiang Province, China [23].

Previous studies showed that the CAP-AB strains are usually sensitive to most antimicrobial drugs [5]. The *A. baumannii* strain, YC128, isolated from the CAP patient, also susceptibility to all tested drugs. WGS results further revealed that YC128 harbored only two β-lactamase genes (blaADC-25 and blaOXA-91). On the contrary, the antimicrobial susceptibility testing showed that *A. baumannii* YC156 was a typical HAP-AB, which was a Carbapenem-Resistant *A. baumannii* (CRAB) strain, resistant to most of the antimicrobial drugs, except tigecycline and colistin. WGS showed YC156 harbored of 16 AMR genes, including β-lactamase genes, Sulfamide resistance genes, and Aminoglycoside resistance genes ect. The drug resistance phenotype and genotype of these two strains are consistent.

The presented results clearly indicate that CAP-AB YC128 is significantly more virulent than HAP-AB YC156 and slightly more virulent than the hypervirulent standard strain LAC-4. When immune-competent mice were infected with HAP-AB YC156, no deaths occurred after inoculation, regardless of whether they received 10^8^ or 10^7^ CFU bacterial suspension. Following 10^7^ CFU HAP-AB inoculation, the pulmonary bacterial counts of the YC156 group remained lower (10^4^ CFU/g tissue) at 24 h and 48 h, while the liver and spleen bacterial burden remained at 0 CFU/ml throughout the process. Previous publications have shown that almost all hospital-acquired *A. baumannii* strains infecting immune-competent mice can only induce self-limiting pneumonia [25, 26].

However, all mice inoculated with 10^8^ CFU YC128 and LAC-4 bacterial suspension died within 48 h. When 10^7^ CFU bacterial suspension was used, the mortality rate was 60% in the YC128 group and 30% in the LAC-4 group. At 24 h post inoculation, the pulmonary bacterial burden was 10^7^ CFU/g tissue for YC128 and 10^5^ CFU/g tissue for LAC-4, and this pattern was consistent at 48 h. The liver bacterial burden of YC128 was slightly higher than that of LAC-4 at both time points, while the spleen bacterial count of YC128 was significantly higher than that of LAC-4 at 48 h. These results confirm that, in contrast to HAP-AB YC156, CAP-AB YC128 is a highly virulent *A. baumannii* strain capable of inducing fatal pneumonia and extrapulmonary dissemination. Moreover, the virulence of CAP-AB YC128 surpasses that of the hypervirulent standard strain LAC-4.

Histopathological tests and CT scans revealed notable differences between HAP-AB YC156 and CAP-AB YC128 infected mice. In the YC156 group, histopathology changes only showed moderate edema in the pulmonary interstitium and a slight inflammatory cell invasion. On the other hand, YC128 group exhibited pathological images depicting lung consolidation, necrosis, furthermore, alveolus and bronchus destruction at 48 h and 72 h post inoculation, accompanied by extensive inflammatory cell infiltration in pulmonary cavity. The more severe CT scan changes observed in YC128-infected mice aligned with the pathological alterations identified in HE-staining. In the YC156 group, consolidation was only evident in the left lung at 48 h, followed by prompt alleviation and returned to normal level at 72 h. In contrast, the CT scans of YC128 group demonstrated a continuous deterioration of image changes, with severe consolidation appearing in bilateral lungs at both 48 h and 72 h. Collectively, the histopathological changes and CT scan results suggest that YC128 can induce severe pulmonary alterations at both histopathological and imaging levels, potentially leading to the death of infected mice due to this critical pneumonia and subsequent bacteremia.

Cytokine/chemokine of IL-1β, IL-6, TNF-α and CXCL1 in the lungs were significantly higher in YC128-infected mice compared to those in YC156-infected mice. The data suggest that the highly virulent CAP-AB YC128 could induce the expression of proinflammatory cytokine/chemokine responses rapidly and severely. Elevated concentrations of these cytokine/chemokine lead to extensive infiltration of inflammatory cells, inducing excessive inflammatory reaction and lung tissue injury, ultimately resulting in lung tissue destruction and consolidation.

To gain a better understanding of the interactions between two *A. baumannii* isolates and the host, we compared the in vitro uptake abilities of *A. baumannii* strains by macrophages. Our results showed that YC128 exhibited greater resistance to macrophage phagocytosis compared to the YC156 strain, implying that an enhanced anti-phagocytosis ability is at least one of the mechanisms contributing to increased virulence of the YC128 strain.

Upon comparing the virulence-related genes of the two strains, the results revealed that YC128 harbored a complete T6SS gene and T6SS secreted effectors gene, whereas only one gene in the T6SS gene cluster was detected in YC156. As a result, YC128 can be categorized as a T6SS-active clinical strain, while YC156 lacks T6SS presence. T6SS represents a class of macromolecular secretion machines that share structural and mechanistic similarities with intracellular membrane-attached contractile phage tails. This syringe-shaped structure is commonly employed by bacteria to inject toxic effectors into host cells [27]. In recent years, an increasing body of research has illuminated the role of T6SS in *A. baumannii* pathogenicity and inter-bacterial competition [28, 29].

Wang et al. demonstrated the necessity of T6SS for *A. baumannii* host colonization using the Galleria mellonella model. Mutant *A. baumannii* strains lacking T6SS components, specifically vgrG, exhibited reduced growth rates, diminished adherence to eukaryotic cells, and decreased lethality in mice [16]. Previous studies have also indicated that T6SS can reduce the phagocytic capacity of macrophages in various Gram-negative bacteria. For instance, in *Burkholderia cenocepacia*, T6SS disarms Rho-type GTPases, leading to actin cytoskeletal defects in macrophages, thereby facilitating the survival of the bacterium within these cells [30]. Additionally, T6SS enables Enterohemorrhagic Escherichia coli (EHEC) to thrive within macrophages by reducing intracellular reactive oxygen species (ROS) levels [31]. Based on previous research findings, it is plausible to speculate that the heightened virulence and robust anti-phagocytosis ability exhibited by YC128 are linked to the expression of T6SS. Nonetheless, further investigations are necessary to determine whether the complete T6SS gene cluster indeed underlies the hypervirulence and anti-phagocytosis capability of *A. baumannii*.

In summary, we present the characterization of a novel CAP-AB strain, YC128, which caused severe pneumonia in an otherwise healthy male patient. The study findings have demonstrated that the CAP-AB YC128 strain possesses the ability to induce fatal pneumonia and extrapulmonary dissemination in a mouse pneumonia model. Furthermore, this highly virulent CAP-AB YC128 strain is also capable of triggering intense pro-inflammatory cytokine and chemokine expressions, along with a notably stronger anti-phagocytosis capacity compared to the HAP-AB strain YC156. WGS analysis revealed that YC128 carries a complete T6SS gene cluster, as well as T6SS secreted effector genes. These findings suggest that the expression of T6SS may be responsible for the heightened virulence and enhanced anti-phagocytosis capability observed in *A. baumannii*.

## Materials and methods

### Bacterial isolates and antimicrobial susceptibility testing

A wild-type strain CAP-AB strain YC128 was isolated from blood culture of the CAP patient in Yichun Hospital, Jiangxi Province, China in 2021. Another wild-type strain HAP-AB strain YC156, from blood culture, was isolated from a ventilator-associated pneumonia (VAP) patient in the intensive care unit (ICU) in the same hospital in 2021. These two strains were further identified by the Vitek GNI card (BioMe’rieux, Marcy-l’Etoile, France) as members of the *Acinetobacter calcoaceticus-A. baumannii* complex. Antimicrobial susceptibility testing was performed by agar dilution method in accordance with the guidelines of the Clinical and Laboratory Standards Institute (2020) [32]. LAC-4, a hypervirulent standard strain, is a gift from Hong-Yu Ou, Shanghai Jiaotong University, Shanghai, China,

### Mice

Age matched female ICR mice were purchased from Slack laboratory animal company (Shanghai, China), weighing from 21 to 25 g. The animals were housed in ventilated micro iso-cages to decrease the risk of extraneous infection. Food and water were provided with no restriction.

### Preparation of *A. baumannii*

One fresh colony from medium culture was inoculated into 20 ml LB broth (Invitrogen, USA) and incubated in 37℃ incubator shaker (150 rpm; New Brunswick Scientific) for 16 h. Then, 5 ml of bacterial suspension was centrifuged at 4000 × g and 4℃ for 10 min, the supernatant was discarded, the residual was then resuspended in 1 ml 0.9% sterile saline. The final bacterial concentration was between 2.0–5.0 × 10^9^ CFU/ml, and then diluted 20 times to a concentration of 1.0–2.5 × 10^8^ CFU/ml bacterial suspension for later use. Actual inoculums concentration was determined by plating tenfold serial dilutions on LB agar plates.

### Mice pneumonia model and mortality of different *A. baumannii* strains

A mice pneumonia model was established as described previously [10]. Briefly, a total of 65 mice were randomized into 7 groups, six groups with each group of 10 mice and saline control group with 5 mice. All mice were anesthetized by a single intraperitoneal injection of 1.25% 2,2,2-tribromoethanol (Sigma-Aldrich, Shanghai, China) at a dosage of 25 mg/kg of body weight. Subsequently, 60 mice were intranasally inoculated with *A. baumannii* in 50 μl of saline, with each group 10 mice. In addition, five mice were intranasally inoculated with 50 μl saline as control. The mice in group YC128(H), LAC-4(H) and YC156(H) were inoculated 10^8^ CFU *A. baumannii* respectively. The mice in group YC128(L), LAC-4(L) and YC156(L) were inoculated 10^7^ CFU *A. baumannii* respectively. The observation time lasted 72 h, and the number of dead mice was recorded every 12 h.

### Bacteria quantification of lung, liver and spleen

To compare the difference of bacterial burden of mice that infected with YC128, LAC-4 and YC156 *A. baumannii* strains, a total of 24 mice were randomized into 3 groups, with each group containing 8 mice. In addition, 4 mice were intranasally inoculated with 50 μl saline as control. The bacterial loads of the inoculants were 5.0 × 10^7^ CFU for YC128, 4.5 × 10^7^ CFU for LAC-4, and 3.2 × 10^7^ CFU for YC156. To obtain tissue for *ex-vivo* studies, 4 mice were euthanized by cervical dislocation after carbon dioxide suffocation at 24 h and 48 h after inoculation in each group respectively. The lungs, liver and spleen from four mice were aseptically collected for bacterial quantitative culture. Briefly, the lungs, liver and spleen of the sacrificed mice were aseptically removed and homogenized in sterile saline, then, the samples (100 μl) of tenfold serial dilutions and cultured on LB plates to quantify the doses of *A. baumannii*.

### Histopathology and CT scan

To compare the difference of pathology and CT scan of mice that infected with YC128 and YC156 strains, a total of 40 mice were randomized into 2 groups, with each group containing 20 mice. One group was inoculated with 10^6^ CFU YC156 and another group with 10^6^ CFU YC128. Five mice were sacrificed at 0 h, 24 h, 48 h, and 72 h after inoculation in each group respectively. The lungs from five mice were aseptically collected for cytokine measurement and one for histopathology. The rest of the lung tissues were fixed immediately in 10% neutral buffered formalin and processed by standard paraffin embedding methods for histopathological examination. The lung tissues were sectioned into 4-μm thick, stained with hematoxylin–eosin (HE), and examined under microscope (Department of Pathology, Fudan University, Shanghai, China). The last sacrificed mouse of each group underwent chest CT scan to investigate changes in the lungs (Department of Radiology, Huashan Hospital, Shanghai, China).

### Determination of cytokine levels

To compare the immunopathogenesis of two isolates, the cytokines levels of the mice lungs, IL-1β, IL-6, TNF-α and CXCL1 in mice pneumonia model were determined by RT-qPCR. Briefly, total RNA was extracted from mice lung tissue using RNA isolation reagent (Pufei, Shanghai, China) according to Trizol RNA isolation protocol. Then, the RNA was transcribed into cDNA using M-MLV Reverse Transcriptase (Promega, Madison, WI, USA) and the cDNA was amplified with AmpliTaq gold (Applied Biosystems, Foster City, CA, USA). The normalization gene was 36B4. The qPCR primers used in this experiment were listed in Table 2.

**Table 2:** Primers used in this study

Target gen	Forward primer(5’ → 3^,^)	Reverse primer(5’ → 3^,^)	Sequence length(bp)
IL-6	CTGCAAGAGACTTCCATCCAG	AGTGGTATAGACAGGTCTGTTGG	131
IL-1β	GAAATGCCACCTTTTGACAGTG	TGGATGCTCTCATCAGGACAG	116
CXCL-1	GCACCCAAACCGAAGTCATA	TGGGGACACCTTTTAGCATC	111
TNF-α	TCCTCACCCACACCGTCAG	GCTGAGTTGGTCCCCCTTCT	176

### In vitro anti-Phagocytosis ability against *A. Baumannii* of macrophages

To further characterize the interaction between two *A. baumannii* isolates and the host, we examined the in vitro anti-phagocytosis ability of *A. baumannii* against mice peritoneal macrophages. Peritoneal macrophages isolated from 5 mice were prepared by using a standard protocol from Davies’ article [33]. In brief, the mice were injected intraperitoneally with 1.5 ml of sterile 3% thioglycollate (Thermofisher, Shanghai, China) per mouse. Three days after injection, the macrophages were harvested from the peritoneal cavity by lavaging with sterile phosphate-buffered saline (PBS). The macrophages were then centrifuged at 4000 g for 10 min and resuspended in Dulbecco's modified Eagle medium (Invitrogen Corporation, Carlsbad, USA) with 10% heat-inactivated fetal calf serum and penicillin (100U/ml) with streptomycin (100 μg/ml). The macrophages of 5 mice were then distributed into different sterile culture wells of a 96-well plate. Each mouse macrophages were distributed into 6 wells containing 200 μl solution, thus 30 samples in total. After 18 h of incubation at 37 °C, non-adherent cells were washed away, and 80% to 90% confluent adherent macrophages (approximately 0.5 × 10^6^/well) were sustained. The cells were then washed and re-cultured in 200 μl culture medium without antibiotics. Then, 20 μl per well of the bacterial suspension of HAP-AB (3.8 × 10^8^ CFU/ml) and CAP-AB (3.2 × 10^8^ CFU/ml) were added into the cell culture. The MOI of bacterial was 10. The cell culture medium was collected, and the suspension was cultured on LB agar plates for 18 h to quantify the extracellular bacterial number after 2 h. Thereafter, the wells were washed using ice-cold PBS for three times, then deionized sterile water was added to the wells to lysis macrophage and release *A. baumannii*. Two hours later, the suspension was cultured on LB plates for 18 h to quantify the intracellular number of *A. baumannii*.

### Whole genome sequencing comparision between YC128 and YC156

The genomic DNA of YC128 and YC156 was extracted and then sequenced using the combination of the 150-bp paired-end Illumina NovaSeq 6000 platform and the PacBio RSII single-molecule long-read sequencing platform. The trimmed and filtered reads were de novo assembled using Canu 2.0 [34]. The genomic data were annotated with Prokka 1.1.3.

Determination of antimicrobial resistance (AMR) performed using VRprofile 2.0 [35]. Virulence genes were identified using the VF Analyzer part of the Virulence Factors of Pathogenic Bacteria (VFDB) database using the defaults settings [36]. We use R packages, ComplexHeatmap and circlize, to visualize genome coverage and add the annotations appropriately.

### Statistical analysis

Differences in mortality rate of groups were assessed by the standard Mentel-Cox logrank test. The survival curves comparision of groups were assessed by the Log rank test. Differences between mean values of two groups normally distributed data including bacterial counting, cytokines expression ect. were assessed by an unpaired Student’s t test. GraphPad Prism 8.0 was used to make figures and perform statistical analysis.

## Data Availability

No datasets were generated or analysed during the current study.

## Abbreviations

CAP: Community-Acquired Pneumonia
HAP: Hospital-Acquired Pneumonia
CAAP: Community-Acquired *Acinetobacter baumannii* Pneumonia
CAP-AB: Community-Acquired Pneumonia *Acinetobacter baumannii*
HAP-AB: Hospital-Acquired Pneumonia *Acinetobacter baumannii*
MIC: Minimum inhibitory concentration
CRAB: Carbapenem-resistant *Acinetobacter baumannii*
CLSI: Clinical and Laboratory Standards Institute.
WGS: Whole genome sequencing
T6SS: Type VI secretion system
